# Synergistic sequential oxidative extraction for nanofibrillated cellulose isolated from oil palm empty fruit bunch

**DOI:** 10.1371/journal.pone.0299312

**Published:** 2024-06-06

**Authors:** Mastura Abd Manaf, Shuhaida Harun, Jamaliah Md. Jahim, Mohd Shaiful Sajab, Zulkifli Ibrahim

**Affiliations:** 1 Faculty of Engineering and Built Environment, Research Centre for Sustainable Process Technology (CESPRO), Universiti Kebangsaan Malaysia, UKM Bangi, Selangor, Malaysia; 2 Faculty of Engineering and Built Environment, Chemical Engineering Programme, Universiti Kebangsaan Malaysia, UKM Bangi, Selangor, Malaysia; 3 Faculty of Electrical and Electronic Engineering Technology, Electrical Engineering Technology Department, Universiti Teknikal Malaysia Melaka, Melaka, Malaysia; The Energy Resources Institute (TERI), INDIA

## Abstract

This research presents a comprehensive study of sequential oxidative extraction (SOE) consisting of alkaline and acidic oxidation processes to extract nanocellulose from plant biomass. This proposed process is advantageous as its operation requires a minimum process with mild solvents, and yet successfully isolated high-quality nanofibrillated cellulose (NFC) from raw OPEFB. The SOE involved ammonium hydroxide (NH_4_OH, 2.6 M) and formic acid (HCOOH, 5.3 M) catalyzed by hydrogen peroxide (H_2_O_2_, 3.2 M). This approach was used to efficiently solubilize the lignin and hemicellulose from Oil Palm Empty Fruit Bunch (OPEFB) at the temperature of 100°C and 1 h extraction time, which managed to retain fibrous NFC. The extracted solid and liquor at each stage were studied extensively through physiochemical analysis. The finding indicated that approximately 75.3%dwb of hemicellulose, 68.9%dwb of lignin, and 42.0%dwb of extractive were solubilized in the first SOE cycle, while the second SOE cycle resulted in 92.3%dwb, 99.6%dwb and 99.8%dwb of solubilized hemicellulose, lignin, and extractive/ash, respectively. High-quality NFC (75.52%dwb) was obtained for the final extracted solid with 76.4% crystallinity, which is near the crystallinity of standard commercial NFC. The proposed process possesses an effective synergy in producing NFC from raw OPEFB with less cellulose degradation, and most of the degraded hemicellulose and lignin are solubilized in the liquor.

## Introduction

The Sustainable Development Goals (SDGs), which were introduced in 2015, are receiving increased attention globally. SDG-2 calls for agriculture to become sustainable, thus reducing, reusing, and recycling the inputs and outputs from agricultural activity [1]. Hence, the research project being reported here uses agricultural residue as a starting material for producing highly useful cellulose nanofibrils. The extraction of cellulose and nanocellulose from different agricultural residues is becoming an important research topic because of its versatile applications despite reduce the dependence on fossil fuels, which is a major contributor to greenhouse gas emissions. It can also help in the development of rural areas by providing a side source of income for farmers as the agricultural waste can be turned into valuable materials. Agricultural residues such as soybean straw [2], sugarcane bagasse, top/leave [3], corn husk/stover/cob/stalks [4,5], oil palm residues [4,6,7], rice straw, wheat straw [4], are among the most suitable residues to be used as cellulose feedstock.

Researchers discovered several advantages of cellulose fiber from oil palm empty fruit bunch (OPEFB), particularly, high specific strength, low density, low cost[8] good electrical and thermal insulation properties, and renewability [9]. So far, OPEFB fiber has been exploited to be used as biodegradable reinforcing thermoplastics and thermosets substance [10], composite polymer to produce micro-perforated panels, particle board, fibers board [11], and thermal insulation panel [7]. The OPEFB cellulosic biomass fiber is creating a revolution as it can be found in various sizes classified as macroscopic, microfibril, nanofibril, and nanocrystal cellulose, depending on the extraction technique [12,13].

Macroscopic cellulose is normally delignified lignocellulose or cellulose pulp with a length of more than 100 μm and a diameter of more than 100 nm [14]. Microfibrillated cellulose (MFC) and nanofibrillated cellulose (NFC) consist of crystalline and amorphous cellulose domains, which contribute to the morphology of soft and long chains. The length of MFC and NFC is 0.5–100 μm and the diameter varies from 20 to 100 nm for NFC and more than 100 nm for MFC, depending on the source of cellulose and defibrillation process [15,16]. Nanocrystalline cellulose (NCC) is stiff rod-like particles consisting of cellulose chain segments in a nearly perfect crystalline structure, with a diameter between 2 and 20 nm while the length varies between 100 and 500 nm [16].

Nanocellulose has favorable properties; water-insoluble biopolymer [17], high specific surface area that typically exceeds 30 m^2^g^-1^ [18,19] high crystallinity, high stiffness up to 220 GPa, low density, and ability to modify its surface chemistry [20]. In addition, it also is full of reactive surface hydroxyl groups that can be functionalized to various surface properties. Due to its versatile morphologies, nanocellulose is used as a reinforcing agent in composite/polymer materials; a continuous fiber for textile, pulp, and paper industries; and a rheology modifier of various media in many industrial applications, such as paints, coatings, adhesives, lacquers, food, cosmetics, drugs, and cement [21].

Common nanocellulose extraction from lignocellulosic biomass involves removal of non-cellulosic components from the feedstock by pretreatment process follow with isolation of nanocellulose from the cellulose [20,22]. In pretreatment, the use of chlorite produces chlorine radicals that react with lignocellulose and subsequently create toxic organochlorine [23,24]. Meanwhile, the utilization of sodium hydroxide (NaOH) causes the formation of salts upon neutralization, which may pose challenges with its disposal during the purification of products [25]. Extraction of nanocellulose from treated biomass by mechanical approaches such as high-pressure homogenization [26], high intensity ultrasonication [27], and steam explosion [28] are environmentally friendly with less chemicals used, however associated with high energy consumption [29] and has limited influence on the chemical structural changes of lignocellulose [30,31]. On the other hand, from the perspective of chemical methods, concentrated acid hydrolysis (8–10 M) to extract nanocellulose can be costly and hazardous due to its toxicity and corrosiveness [32]. Therefore, mild and dilute chemical treatment has become an attractive alternative for biomass modification. However, the process was conducted for 13–16 h for nanocellulose extraction, depending on the substrate condition; consequently, the method resulted in a low extraction yield of nanocellulose in the range of 20%–30% [32,33].

**Fig 1:**
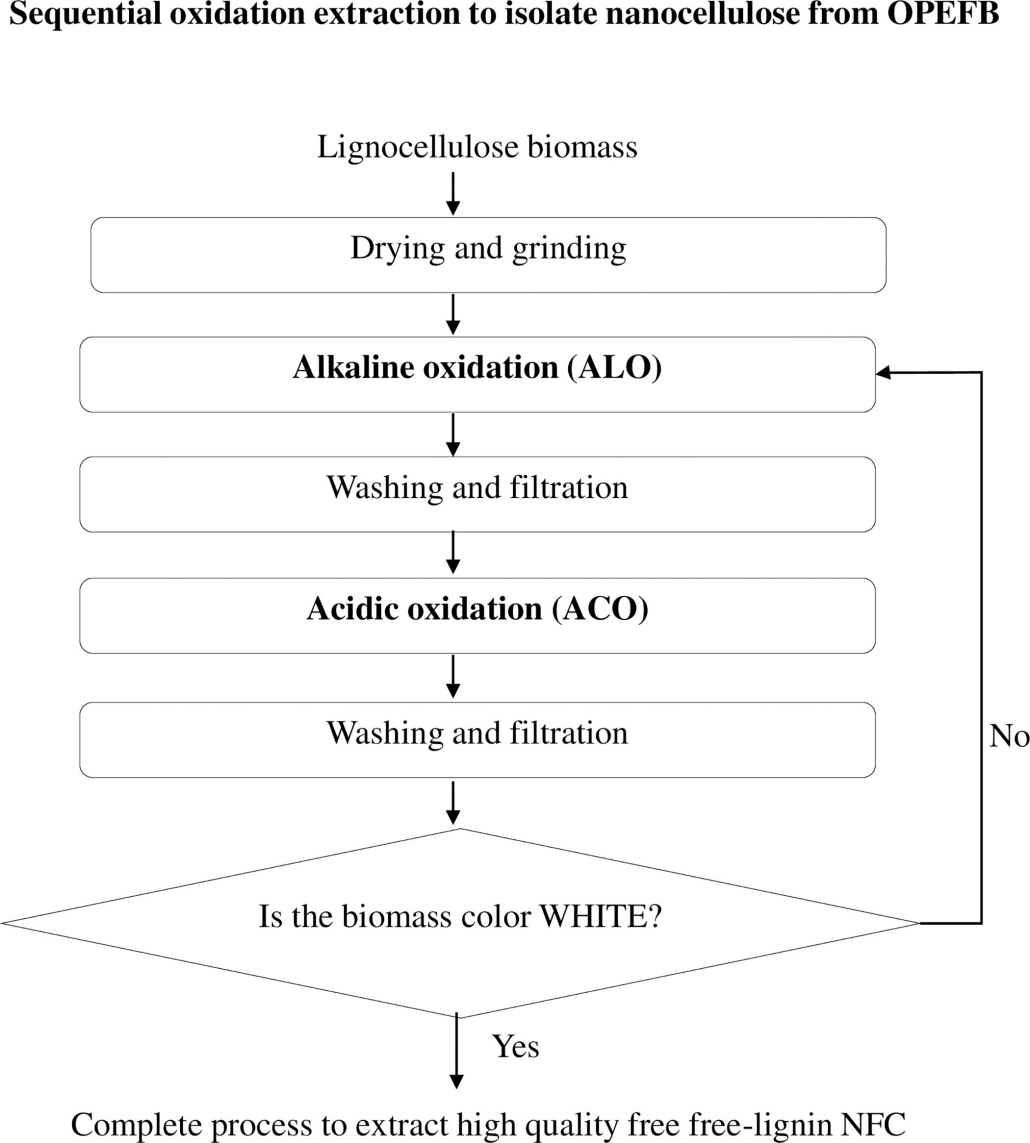
Process flow of sequential oxidative extraction for nanofibrillated cellulose isolated from oil palm empty fruit bunch.

Thus, this study proposed to explore the potential of mild base-acid treatment catalyzed by hydrogen peroxide (H_2_O_2_) oxidation to extract the nanocellulose from OPEFB. The oxidative process acts in the delignification and solubilization of biomass [34–36] and H_2_O_2_ could significantly shorten 70% of the processing period from 6 h to 2 h and increase the cellulose composition from 30 wt. % to 95 wt. % [35,36]. The alkaline oxidation (ALO) and acidic oxidation (ACO) involve the use of H_2_O_2_ in different pH conditions by alkaline (ammonium hydroxide) and acid (formic acid) reagents. Ammonium hydroxide (NH_4_OH) is a weak alkali compared to sodium hydroxide (NaOH), and it preserves the cellulose due to low alkali reactivity with carbohydrate [37,38]. Formic acid, an organic acid, has been used as an agent for delignification since 1917 and the technology known as Milox process. It is also less corrosive, can be recovered easily and effective for biomass fractionation, and provides a more stable medium compared to sulfuric acid [39,40]. The synergistic action of the presented mild ALO and ACO to directly produce nanocellulose from the biomass without other assistant physical step is the novelty that is emphasis in the study as shows in Fig 1. This mechanism shortens the processing time and is also less energy intensive.

## Materials and methods

### Materials

Oil palm empty fruit bunch was collected from Kok Foh Palm Oil Mill, Bahau, Negeri Sembilan. The biomass was sun-dried until its moisture content was approximately 10% of OPEFB dry weight. Then, the biomass was ground into approximately 1–2 cm particle size using a universal cutting mill (Model: Pulverisette 19 Fisch, Germany).

### Chemicals

The analytical grade chemicals used for the sequential oxidative extraction (SOE) of ALO and ACO extraction were ammonium hydroxide (30%–33% NH_3_ in H_2_O, CAS #: 1336-21-6), hydrogen peroxide (30% H_2_O_2_, CAS #: 7722-84-1), and formic acid (90% HCOOH in H_2_O, CAS #: 64-18-6). Meanwhile, the analytical grade chemicals used for the analysis (i.e., chemical composition, sugar content, and inhibitor determination analysis) were D (+)-glucose, D(+)-xylose, L(+)-arabinose, sulfuric acid (H_2_SO_4_, 95%–99%), 5-hydroxymethyl-2-furaldehyde (99%), acetic acid, and furfural, which were acquired from Sigma-Aldrich (M) Sdn. Bhd. (Sigma-Aldrich, St. Louis, MO). Two celluloses were used as comparison in this study, which were Avicel® PH-101, a pure cellulose obtained from Merck (Cellulose powder, CAS#9004-34-6) and industrial produced NFC from plant biomass, purchased from Waris Nove (M) Sdn Bhd, Kuantan, Pahang.

### Cellulose extraction by sequential oxidative extraction (SOE)

The prepared OPEFB feedstock was subjected to SOE, starting with ALO and followed by ACO. The extracted solid after the first SOE undergoes another cycle of SOE until a whitish solid was obtained. The in-process water washing between these oxidative treatments was used to remove the solvent and brownish/yellowish lignified effluent from the sample. The extracted solids and liquors were collected for further analysis.

#### Alkaline oxidation (ALO)

About 15 g in dried weight basis (dwb) of OPEFB was soaked in alkaline liquor at a solid-to-liquid loading ratio of 1:20. The liquor mixture contained a 1:1 volume ratio of 10% v/v NH_4_OH (2.6 M) and 10% v/v H_2_O_2_ (3.2 M). The mixture was heated to 100°C and mixed at 100 rpm for 60 min. After that, the sample was left for another 20 min to let the solid biomass cool. Then, the supernatant liquid was separated from the biomass fiber using a polyester filter cloth and washed with distilled water at 25˚C temperature (volume of water same as volume of solvent used in the process). Weight and moisture content of the solid fiber was recorded to measure the yield of remaining solid in dwb. Subsequently, the alkaline-pretreated OPEFB was subjected to ACO.

#### Acidic oxidation (ACO)

Alkaline-oxidized solid fiber was soaked in solid-to-liquid loading ratio of 1:20. The liquid was composed of 20% HCOOH (5.3 M) and 10% H_2_O_2_ (3.2 M) at a 1:1 volume ratio (v/v). The mixture was heated to 100°C and mixed at 100 rpm for 60 min. The fibrous solid was separated from the liquid supernatant, washed using distilled water at 25˚C temperature (volume of water same as volume of solvent used in the process), and filtered using a polyester filter cloth. Weight and moisture content of the solid fiber was recorded to measure the yield of remaining solid in dried weight basis (dwb). At this stage, the extracted solids (ES) were subjected to a cycle of ALO and ACO until a whitish solid was obtained. The remaining solid obtained after the respective ALO and ACO pretreatment was determined using Eq (1).


$$Yield\;of\;remaining\;solid\;(\%)=\left(\frac{mass\;ES\;after\;each\;process\;(g\;dwb)}{mass\;raw\;OPEFB\;(g\;dwb)}\right)\times 100$$ (1)

### Chemical composition analysis

#### Chemical composition analysis of solid fraction

Chemical composition of raw OPEFB, ALO, and ACO solid fibers was evaluated using Laboratory Analytical Protocols from the National Renewable Energy Laboratory (NREL, Colorado) [41]. The protocols referred to are the Preparation of Samples for Compositional Analysis (NREL/TP-510-42620) and Determination of Structural Carbohydrate and Lignin in Biomass (NREL/TP-510-42618). All samples (n = 3 for each type of sample) were extracted with distilled water and 95% ethanol using an accelerated solvent extractor (Dionex ASE 350, Thermo Scientific, Sunnyvale, CA) for extractive removal prior to quantifying the structural carbohydrates and lignin. The samples undergo double hydrolysis, first with 72% H_2_SO_4_ (shacked and incubated in 30˚C for one hour) then water was added to dilute the acid to 4% before autoclaved at 121˚C. The samples were let to cool prior to filtrate and inject into the high-performance liquid chromatography (HPLC) system (Thermo Scientific UltiMate 3000 LC, Dionex, Sunnyvale, CA) using a Rezek ROA column (Phenomenex) as the stationary phase. The system was equipped with a refractive index (RI) detector (RefractoMax 520, ERC, Germany) set at 60°C, a Rezex ROA organic acid column (300 mm × 7.8 mm; Phenomenex, USA), a guard column (50 mm × 7.8 mm), and 0.005 N H_2_SO_4_ as the mobile phase (isocratic elution, single mobile phase composition). Calibration curves and sample accuracy were performed using software Chromeleon v. 7.2.2.6686 (Dionex, Sunnyvale, CA). Acid-insoluble lignin analysis was performed by referring to the standard method [42]. Moisture and ash content were determined using a moisture analyzer and a furnace [43]. The composition data of ES upon each process were further analyzed to determine the effect of ALO and ACO on cellulose, hemicellulose and lignin composition.

#### Chemical composition analysis of liquid fraction

The chemical composition of the residue liquor and washing effluent generated from SOE was analyzed using HPLC. The samples were hydrolyzed, filtered, and injected into the HPLC system (Thermo Scientific UltiMate 3000 LC, Dionex, Sunnyvale, CA) using a Rezek ROA column (Phenomenex) as the stationary phase system with an RI detector (RefractoMax 520, ERC, Germany) and a guard column (50 mm × 7.8 mm). Degassed 5 mM H_2_SO_4_ was used as the mobile phase at a flow rate of 0.6 mL/min and a column temperature of 60°C. The HPLC sample injection volume in both analyses was 10 μL. Calibration curves and sample accuracy were performed using software Chromeleon v. 7.2.2.6686 (Dionex, Sunnyvale, CA) [42,44]. The liquid chemical composition was presented as the weight of component in liquor fraction over the weight of components in input solid.

Quantification of acid-soluble lignin in all liquid streams was calculated based on the absorbance value at 280 nm using ultraviolet-visible (UV-Vis) spectroscopy (Beckman DU Spectrophotometer, 600 series). Duplicate sample aliquots with a volume of 10 mL were added to 2 mL of 6.5 M NaOH and diluted with deionized water to produce a final volume of 100 mL and a pH of approximately 12.0. The sample absorbance was set at 280 nm using a UV-Vis spectrophotometer. The acid-soluble lignin content was determined according to the absorptivity value and the dilution ratio (10×) and calculated according to the following expression [45]

$$C_{Slig}=[4.187\times 10^{-2}\;(A_{lig280})]-{3.279\times 10}^{-4}$$ (2)

where *C*_*Slig*_ is the concentration of soluble lignin (g/L) and *A*_*lig*280_ is the absorbance of the solution sample at 280 nm.

### Fourier transform infrared spectroscopy (FTIR)

The functional groups of cellulose fibers were analyzed using a Nicolet 67000 FTIR spectrometer (Thermo Fisher Scientific, Waltham, MA). ATR was used without any extra materials or holder. All sample spectra were scanned in the range of 4000–600 cm^-1^ under a wavelength resolution of 4 cm^-1^ and recorded in transmission mode [25]. Then, the FTIR analysis was used to determine the lignin-to-cellulose ratio. The band area at 1504–1507 cm^-1^ is assigned to the total content of lignin (aromatic skeleton vibration) and the band area at 1371–1372 cm^-1^ represents the C-H bond in cellulose [46]. Furthermore, ten peaks related to lignocellulose functional groups were identified from the spectra at wavelengths ranging from 3500 to 899 cm^-1^ and labeled as Peak A to J (Table 4). Peak intensity strength was compared between samples based on the peak height ratio. For example, as Peak A located at 3222 cm^-1^, the peaks height for each sample at that peak was checked and compared.

#### Nuclear magnetic resonance (NMR) analysis

The cellulose fiber was further analyzed for its structure by NMR analysis. The ^1^H NMR measurements were performed on Bruker Avance AV III HD-400 MHz spectrometer (MA, USA). The samples were prepared in DMSO (30 mg of sample dissolved in 0.6 mL DMSO). A 90˚C pulse flipping angle, a 1–4 second acquisition time and a 1–2 second delay time between scans were set in the analysis.

### Characterization of solid fiber

#### Morphology analysis

The morphology of native and extracted solids were observed using Field Emission Scanning Electron Microscope (FESEM), model MERLIN, brand ZEISS, under 15 kV acceleration voltage. The solid sample was also analyzed with Transmission Electron Microscope (TEM), brand Talos L120C under resolution 120kV for imaging the internal structure of the sample. 10 μL drop of 0.1 wt. % cellulose dispersion was mounted on a carbon-coated TEM copper grid. Excess liquid was blotted with filter paper [35]. The image was processed by using Image J software version 1.53. A minimum of five images were taken and analyzed for each sample. For each sample, ten particle length and diameter data were recorded to analyze the mean and standard deviation.

#### X-ray diffraction (XRD) analysis

The XRD analysis was used to determine the amount of amorphous and crystalline content in an extracted solid sample. All samples were analyzed using D8 Advance XRD (Bruker AXS, Germany) and analysis was carried out with Cu Kα1 radiation source with the wavelength of 0.15406 Å at 40 kV and 40 mA, intensity (2θ) within 20° to 80° at a speed of 0.25° per second. The XRD patterns were recorded at room temperature. The crystallinity index of the samples was calculated as Eq 3.

$$Crystallinity\;index\;(\%)=\frac{I_{002-}I_{am}}{I_{002}}x\;100$$ (3)

where I_002_ (2θ = 22.5°) is the intensity that represents both crystalline and amorphous material and I_am_ (2θ = 18°) is the intensity that represents amorphous material [25].

#### Water absorption

The water absorption number (WAN) of extracted solid fiber was estimated according to ASTM-D570 [46]. The samples were immersed in distilled water. The weights of the tested nanocomposites were recorded until constant weight was obtained. Eq 4 was utilized to calculate the WAN.

$$WAN\;(\%)=\frac{w_{n-W_{i}}}{w_{i}}\;x\;100$$ (4)

where, Wn represents the weight of the sample after immersion and Wi is the weight of the composite before immersion.

#### Zeta potential

The electrokinetic characterization, surface charge of the solid sample after each process involved the measurement of the zeta potential using a Malvern Zetasizer Nano ZS (ZEN 3600, Worcestershire, UK) with the aid of Dispersion Technology Software version 5.02. The concentration of solution was determined in 0.05 mg/mL, at temperature 25˚C and equilibrium time 120 seconds [47].

#### Thermalgravimetric analysis (TGA)

TGA is a technique for the measurement of thermal stability of solid fibers where the weight of sample is measured while its temperature is increased. The analysis was carried out using a Netzsch thermal analyzer with Proteus software. Samples of approximately 6 mg were placed in alumina pans and heated from 30 to 600˚C at 10˚Cmin^-1^ under a dynamic flow of nitrogen (50 mLmin^-1^).

### Statistical analysis

All solid and liquid samples for chemical composition were performed in duplicate (n = 2 for each type of sample). Data was expressed as mean ± standard deviation (SD). Measurements for each analysis were carried out in triplicate (n = 3) for each sample and statistically analyzed using Minitab Statistical software version 17, Stata Corp LLC, USA. One-way Analysis of Variance (ANOVA) was performed followed by comparison post-hoc test using Turkey’s method (95% confidence level, p < 0.05). The P value of less than 0.05 was considered statistically significant.

## Results and discussion

### Sequential oxidative extraction

**Fig 2:**
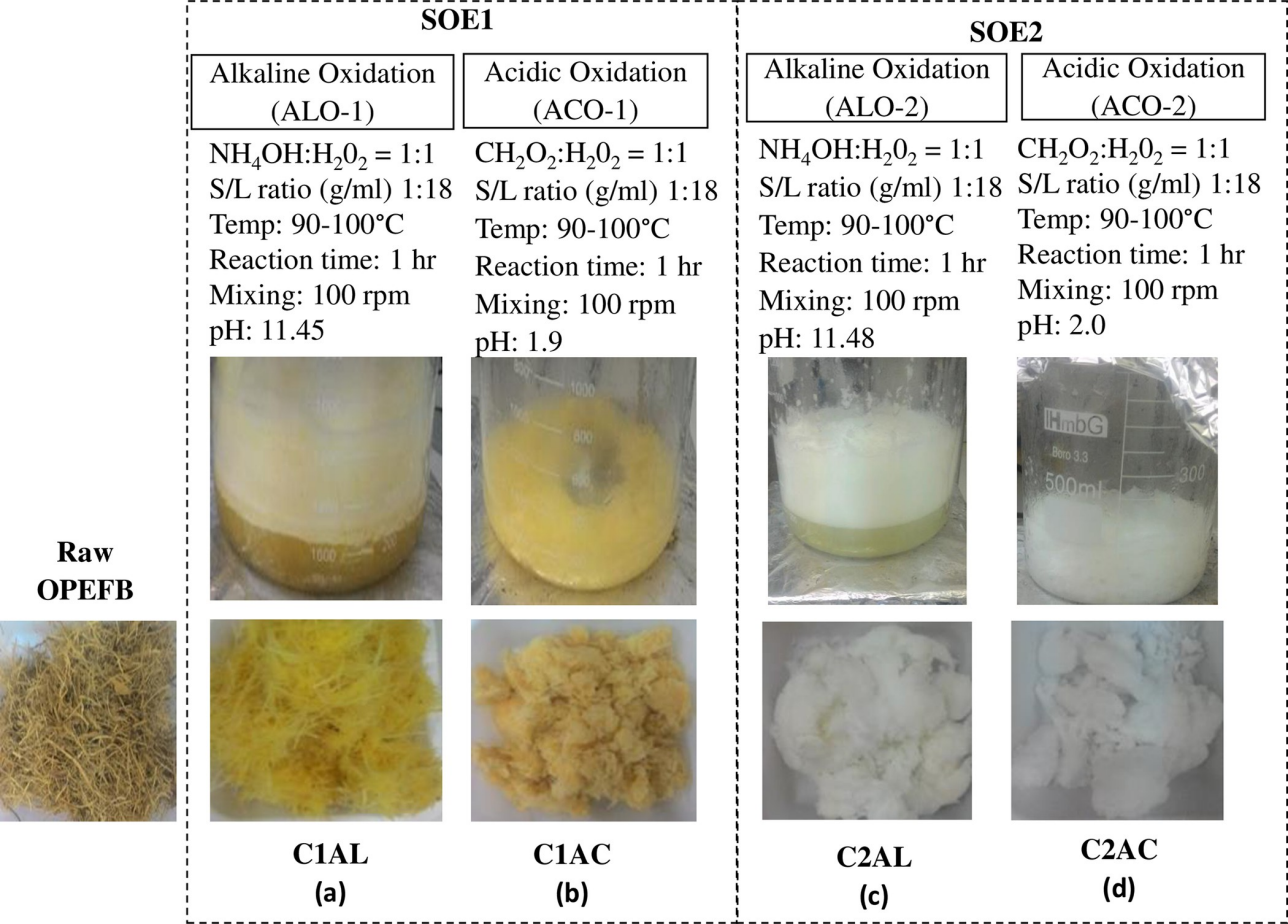
Sequencial oxidation extraction of OPEFB and the extracted solid upon ALO1 (a), ACO1 (b), ALO2 (c) and ACO2 (d).

The prepared OPEFB was subjected to SOE, where the extraction cycle started with ALO and followed by ACO. The ALO process consisted of 0.05% v/v NH_4_OH (over the total working volume of the process) and same concentration of H_2_O_2_. After 1 h of ALO, yellowish extracted solid (ES) was obtained compared to the brownish raw OPEFB, as shown in Fig 2A. Then, the alkaline oxygenated OPEFB was subjected to another hour of ACO in the presence of 0.1% v/v CHOOH (over the total working volume of the process) and 0.05% v/v H_2_O_2_. Upon the completion of ACO in the first SOE cycle (ACO1), the ES sample was observed to be thinner, and the dark yellowish pulp clumped together (Fig 2B), thus the ES was subjected to the second cycle of SOE to obtain finer white fiber. The second cycle SOE was conducted similarly to the first cycle. Upon completion of the second ALO (ALO2), the ES was in light yellow (Fig 2C) and the fiber turned to snow-white colored (Fig 2D) after the second ACO (ACO2). Therefore, two SOE cycles without supplement of physical process are sufficient to be an alternative approach for obtaining white cellulose from the OPEFB biomass. Further analyses were conducted on the extracted solid fiber to determine the characteristics and quality of the cellulosic based on the chemical composition, physical, and morphology analysis.

#### Chemical composition of solid fraction

**Fig 3:**
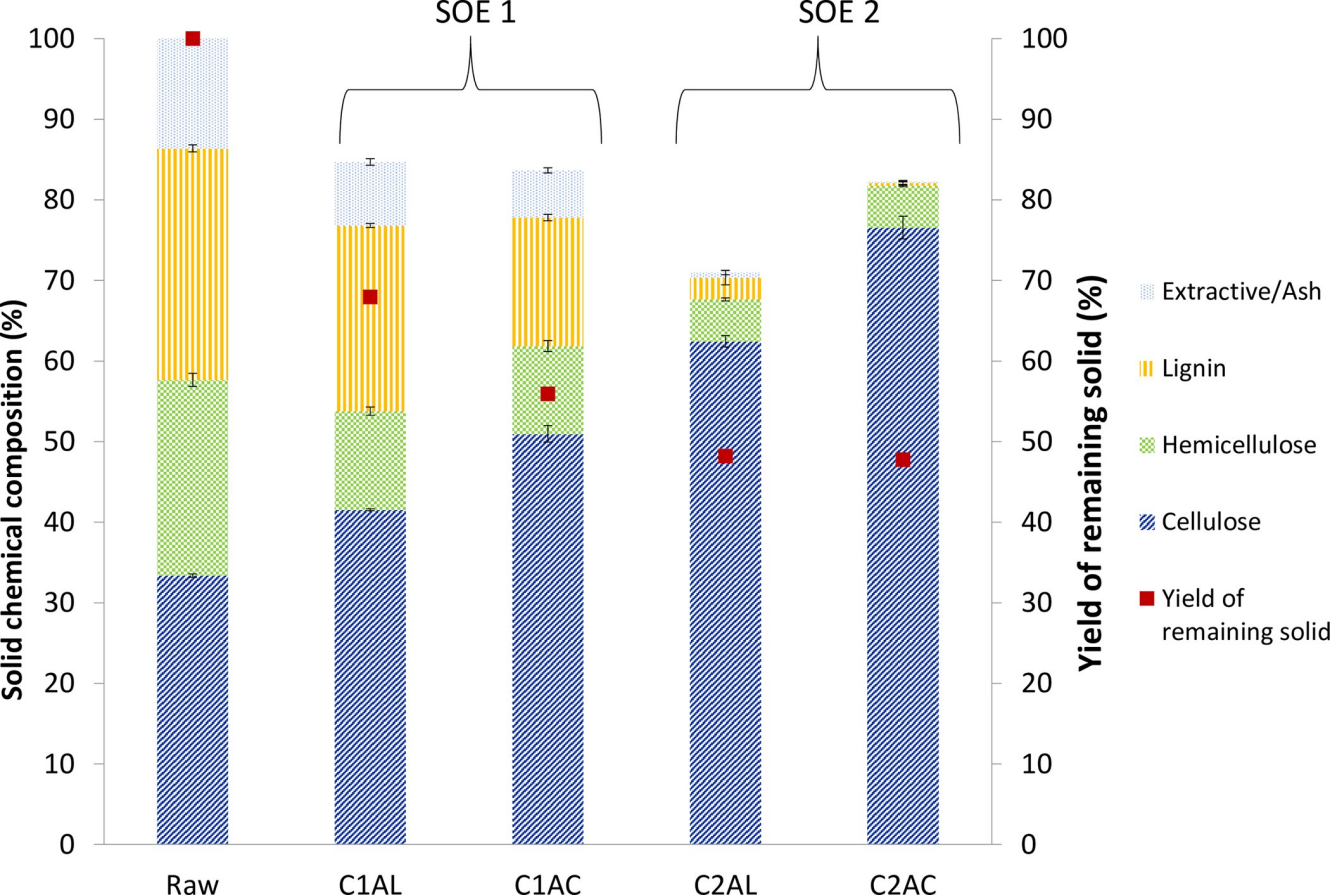
Solid chemical composition of raw and extracted solid upon for each process cycle and yield of remaining solid. Chemical compositions shown in each sample are the mean data (n = 2) with error bars representing standard deviation.

Chemical composition of raw OPEFB (Fig 3) in terms of dry weight percent (w/w), primarily consist of 33.38±1.22% cellulose, 24.29±1.22% hemicellulose,28.72±1.73% lignin, and extractives (14.66±0.72%). The result obtained in this study is congruent with previously published OPEFB composition [41,48–50]. This result also indicates that the major structural constituents of OPEFB are dominated by cellulose (generally referred to as β-1,4-glucan), followed by lignin and xylan as the main components of hemicellulose.

#### First cycle of SOE

Upon completion of the alkaline (ALO-1) and acidic oxidation (ACO-1) in the first SOE cycle, yield of the remaining solid after the extraction was reduced to 67.9% and 55.9% respectively due to solubilization of extractive, lignin, and hemicellulose components from the solid throughout the SOE process. This is verified by solid composition analysis where the hemicellulose composition dropped from 24.29±0.81% in raw OPEFB to 12.25±1.51% in C1AL and 10.9±0.68% in C1AC, while the lignin composition decreased from 28.7±1.73% in raw OPEFB to 23.03±1.84% in C1AL and 15.95±2.38% in C1AC (Table 1). The dissolution of the hemicellulose and lignin from the solid biomass, leading to increase of the cellulose composition from 33.38% to 41.54% in C1AL and 50.96% in C1AC. In the closest method described by Kim, Lee, and Park (2000), newspaper and pulp mill waste were treated in ammonia regenerated percolation (ammonia and hydrogen peroxide at 170˚C), resulted decrease in cellulose fraction from the treated biomass from 53.3 to 45.29% because of an excessively high reaction temperature Nevertheless, the study reported about 30% of the lignin and 50% of hemicellulose are removed from the biomass by the process [51]. The finding showed that, the presented process reaction hindered the cellulose degradation from the solid biomass while the lignin and hemicellulose efficiently dissolved to the liquor stream. However, study on effect of acidic oxidation subsequent of the presented alkaline oxidation treated biomass is very limited. However, study from Li et al. (2016) has reported two stages of treatments with strong alkali and acid (NaOH and H2SO4 at 120˚C) resulted 53% and 26% delignification after alkaline and acid treatment, respectively, along with a 56˚% and 78% elimination of hemicellulose [52].

**Table 1:** Remaining solid yield, chemical composition, fiber dimension and fiber classification of extracted solid C1AL, C1AC, C2AL, and C2AC.

	Extracted solid (ES)			
	C1AL	C1AC	C2AL	C2AC
Remaining solid yield (%)	67.96	55.94	48.18	47.75
Chemical composition (%)				
Cellulose	41.54±0.11^a^	50.96±1.03^b^	62.44±0.72^c^	78.85±4.41^d^
Hemicellulose	12.25±1.51^a^	10.9±0.68^b^	5.21±1.19^c^	5.20±0.14^c^
Total lignin	23.03±1.84^a^	15.96±2.38^b^	2.71±0.91^c^	0.34±0.30^d^
Extractive	7.88±0.41^a^	5.85±0.31^b^	0.61±0.26^c^	0.15±0.03^d^
Fiber dimension (nm)				
Diameter	167.82±21.7^a^	81.69±18.5^b^	40.23±5.5^c^	36.04±6.1^d^
Length	4351.29±99.8^a^	3888.11±43.7^b^	1486.41±328.1^c^	764.31±165.5^d^
Fiber classification	Lignin containing micro-fibrillated cellulose	Lignin containing nano-fibrillated cellulose	Nano-fibrillated cellulose	Nano-fibrillated cellulose

Averages are means of two determinations (n = 2) ± standard deviation.

^abc^ represents the significance difference between samples from one-way ANOVA (Turkey method, 95% confidence). Different letters indicate significantly different between samples.

#### Second cycle SOE

The chemical composition analysis verified that lignin and hemicellulose in the solid fiber were further decreased upon second cycle SOE. Fig 3 shows bigger changes in lignin composition happen in SOE2 which is 2.71±0.91% in C2AL to 0.34±0.3% in C2AC. While hemicellulose composition decreased to 5.21±1.19 in C2AL and remained at 5.20±0.14 in C2AC, less significant changes in hemicellulose composition occurred in the acidic extraction of this cycle. Moreover, the soluble extractive also dropped to 0.61±0.26 (C1AL) and 0.15±0.03 (C1AC) at the end of the process. Due to the increase of lignin dissolution after alkaline and acidic extraction in the second SOE cycle, the cellulose composition increased to 62.44±0.72% (C2AL) and 76.57±4.41% (C2AC). Besides that, Kaur et al., (2023) also reported cellulose composition increments of rice straw from difference protocols. The result showed that alkaline treatment (5% w/w NaOH at 121˚C) followed by bleaching (NaClO2 at 75˚C) had a remarkable efficiency with an increase in cellulose composition from 37.2 to 64.3%. However, the process is less green due to strong alkali and generation of chlorine dioxide. Furthermore, in comparison to greener option, organosolvent treatment (formic acid at 160˚C) and steam explosion (180˚C), these methods are reported increased of cellulose composition from 37.2% in untreated rice straw to 46.5 and 43.7%, respectively [53].

### Material balance for the sequential alkaline and acidic oxidative extraction for free lignin nanofibrillated cellulose from oil palm empty fruit bunch

**Fig 4:**
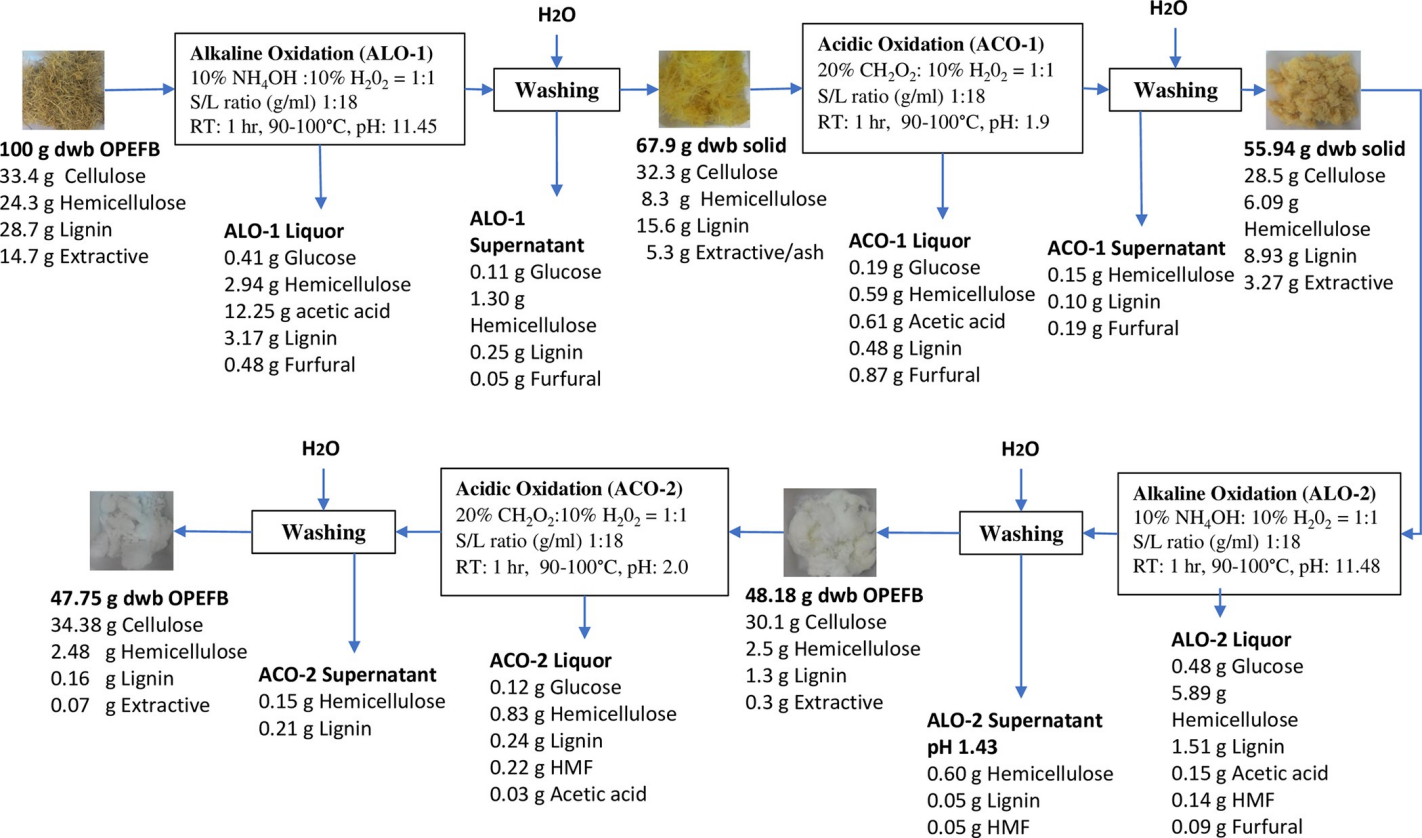
Material balance chemical composition analysis of liquor fraction and sugars degradation upon the ALO1, ACO1, ALO2 and ACO2.

The material balance of solid and liquid fraction of the entire alkaline and acidic oxidation for extraction of the free lignin NFC from OPEFB were computed in a methodical manner as described in Fig 4. Dried 100 g of OPEFB was used as the basis for the material balance of the NFC extraction by two cycle SOE method. The alkaline oxidation (ALO-1) of first SOE resulting 67.96% recovery of solid fraction consisting of 41.5% cellulose while 5.69% of lignin and 8.94% of hemicellulose were dissolved into liquor and wash stream. The acidic oxidation (ACO-1) solubilized 7.08% from remaining lignin and 1.35% from hemicellulose. The remaining solid corresponding to 56% of original material was further subject to SOE2. The second cycle of SOE gave more effect in the lignin dissolution from solid, where 13.24% of lignin while 5.71% hemicellulose were removed out in ALO-2 resulting in 48.2% recovery of solid fraction enriched in cellulose (62.44%). A total of 47.75% solid from the starting biomass was successfully recovered upon ACO-2 consisting of most cellulose, while in total 99.4% lignin and 89.8% hemicellulose were dissolved into the liquid stream.

Soluble lignocellulose components of solid OPEFB degraded during ALO and ACO were transferred into the liquor and wash stream. In the case of soluble glucose (C6 sugar, monomers of cellulose), upon the first SOE cycle, 5.2 mg/g of glucose was solubilized into the ALO-1 liquor, while low glucose content (2.89 mg/g) was detected in the ACO-1 liquor, consistent with lower cellulose degradation in the ACO-1. The same phenomenon (low soluble sugar in the acidic liquor) was observed in the second SOE cycle, where 8.56 mg/g and 2.45 mg/g of soluble glucose were found in the ALO-2 and ACO-2 liquor, respectively. This result found out that the cellulose fraction is more susceptible to alkaline extraction compared to acidic extraction. The presence of glucose in the liquor might be due to the removal of some amorphous components from the fiber structure. In the case of soluble xylan and arabinan (C5 sugar), higher soluble xylan and arabinan were detected in the alkaline liquor than the acidic liquor, with 42.42 mg/g and 8.73 mg/g in the ALO-1 and ACO-1 liquor, respectively. The second SOE cycle recorded soluble xylan and arabinan of 105.33 mg/g and 17.17 mg/g in the ALO-2 and ACO-2 liquor, respectively. The result shows that higher soluble xylan and arabinan are found in the second cycle of ALO and ACO. This is due to continuous solubilization of hemicellulose in the second cycle even though lower hemicellulose content remained in the solid OPEFB for the ALO-2 and ACO-2. However, some amount of HMF was quantified from the ALO-2 and ACO-2 liquor (2.64 mg/g and 4.51 mg/g, respectively), indicating degradation of cellulose in the second cycle. The conversion of C6 components to HMF occurs as cellulose is more exposed to oxidation and hydrolysis processes.

#### Effect of ALO and ACO

**Fig 5:**
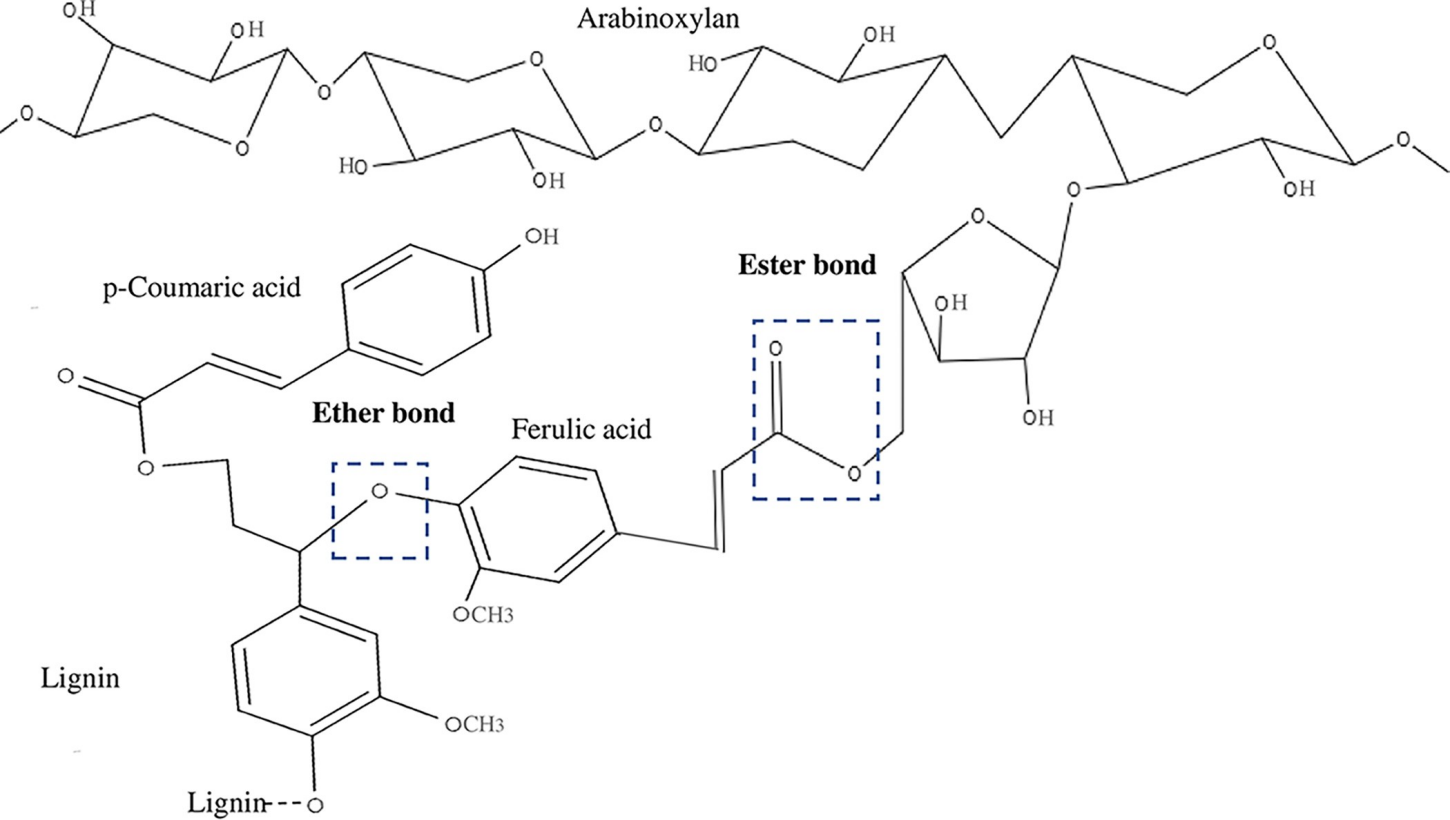
Lignin carbohydrate complex. Redraw and adapted from Singh and Sharma (2020) [55].

Both ALO and ACO contributed to the extraction of nanocellulose from OPEFB either through chemical or catalytic route. The mechanism of the ALO and ACO affected the lignin, hemicellulose and cellulose was simplified at Table 2. In ALO process, hydroxide ions (OH) break the alkaline labile intermolecular ester bond between lignin and hemicellulose through the ferulic acid bridge in the lignin-carbohydrate complex (LCC), as shown in Fig 5. The mechanism of OH ions that react with lignocellulose, involves ester bond cleavage by the alkaline process, leads to the disruption of LCC, swelling, and lignin and hemicellulose solubilization [54]. This mechanism is supported by the functional group analysis by FTIR spectroscopy, particularly the C = O stretching peak at 1750 cm^-1^, which is assigned to the ester groups in the hemicellulose and p-coumaric acid of lignin. This peak intensity reduced and diminished after the ALO-1, indicating the interruption and delignification of the LCC structure [46].

**Table 2:** Effect of ALO and ACO on lignin, hemicellulose, and cellulose.

	ALO	ACO
Effect to lignin	i Hydroxide ions (OH) break the alkaline labile ester bond in LCC ii O2˙ and HO˙ radicals attack at aryl ether bonds of phenolic compounds.	i. Disruption on acid labile ether bond in LCC ii. HCOOH and hydroxonium ion’s reaction toward the aromatic chains in lignin, leading to cleavage of the lignin b-O-4 bonds.
Effect to hemicellulose	Cleavage of ester linkage of hemicellulose with other compounds.	Hydrolysis degrades hemicellulose to monosaccharides such as furfural and acetic acid.
Effect to cellulose	Peeling and stopping reaction shorten the cellulose chain	Low hydrolysis converts crystalline cellulose domain to amorphous.

In addition, as the extraction mixture contains NH_4_OH and H_2_O_2_ with a pH of approximately 11.45, there is a tendency of unstable H_2_O_2_ to undergo bond dissociation, thus generating a strong nucleophile hydro-oxidation anion (HOO-). The hydro-oxidation anion (HOO^-^) reacts with excess H_2_O_2_, leading to the formation of reactive superoxide radical (O_2_˙) and hydroxyl radical (HO˙) as reaction (5–6). The O_2_˙ and HO˙ radicals are reported to be responsible for the attack at aryl ether bonds of phenolic compounds, which explains the formation of phenolic compounds during alkaline treatment and the opening of the phenolic ring of lignin by a nucleophilic attack (Eq 7). Meanwhile, the unreacted radicals disintegrate into O_2_ and H_2_O (Eqs 8 and 9) [56].


$$H_{2}O_{2}↔H^{+}+{\mathrm{HOO}}^{‐}$$ (5)

$$H_{2}O_{2}+{\mathrm{HOO}}^{‐}↔{\mathrm{HO}}^{\cdot }+{O_{2^{‐}}}^{\cdot }+H_{2}O$$ (6)

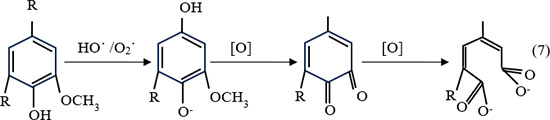



$$2.{\mathrm{OH}}^{\cdot }↔H_{2}O_{2}$$ (8)

$$2H_{2}O_{2}↔O_{2}+H_{2}O$$ (9)

Comparing the ALO mechanism with strong alkali reaction that usually involves NaOH treatment at elevated temperatures (160 to 170˚C). Hydroxide ion (dissociated from NaOH) attacks the carbon of the ester bond between the lignin and the other carbohydrates or between two carbohydrate components, form a tetrahedral intermediate. The deprotonating of the carboxylic acid causes the irreversible hydrolysis of ester bond and weakens the lignocellulose structure [57]. A study compared the pretreatment process of sodium hydroxide and soaking ammonium hydroxide. The results showed that pretreatment with ammonium hydroxide resulted in greater content of cellulose compared to pretreatment with sodium hydroxide pretreatment because ammonia solutions are more selective in eliminating lignin and do not dissolve β-cellulose and γ-cellulose [58]. Furthermore, a bleacher that is commonly in pulp and paper industry, sodium hypochlorite (NaClO) solution play an important role in brightening the pulp slurry by oxidizing and alteration of the hexenuronic acid groups (Hex A) in the pulp during hypochlorite bleaching. However, the process is less green due to corrosive and generation of chlorine dioxide [59].

Upon the ACO, the remaining solid yield continued to decrease. In the presence of HCOOH and H_2_O_2_, ACO contributes to the continuous solubilization of lignin and hemicellulose from the solid OPEFB. As reported by Filippis et al. (2009) and Nada et al. (2003), the following reactions are believed to occur in the medium containing HCOOH and diluted H_2_O_2_ [60,61]: (i) the reversible oxidation of formic acid (HCOOH) by accepting an oxygen atom from H_2_O_2_ that resulted in the formation of peroxyformic acid (HCOOOH) (Eq 10) and the decomposition of HCOOOH generated HCOOH and hydroxonium ions (Eq 11). Electrophilic hydroxonium ions are particularly active towards the olefinic and aromatic chain of lignin. The reaction causes degradation of the high molecular mass of lignin (ii) the irreversible acid-catalyzed decomposition of HCOOOH and (iii) decomposition of H_2_O_2_ in an acidic condition producing water, O_2_, and CO_2_ (Eqs 12–15).


$$H_{2}O_{2}+\mathrm{HCOOH}↔\mathrm{HCOOOH}+H_{2}O$$ (10)

$$H^{+}+\mathrm{HCOOOH}↔\mathrm{HCOOH}+H_{3}O^{+}$$ (11)

$$\mathrm{HCOOOH}\to {\mathrm{CO}}_{2}+H_{2}O$$ (12)

$$2\mathrm{HCOOOH}\overset{H^{+}}{\to }2\mathrm{HCOOH}+O_{2}$$ (13)

$$\mathrm{HCOOOH}+H_{2}O_{2}\overset{H^{+}}{\to }\mathrm{HCOOH}+H_{2}O+O_{2}$$ (14)

$$2H_{2}O_{2}\overset{H^{+}}{\to }2H_{2}O+O_{2}$$ (15)

The dilute acid mixture (20% HCOOH solution with 10% H_2_O_2_ solution) resulted in a solution of pH 1.38. This acidic condition is responsible for fractionating and potentially hydrolyzing lignocellulosic materials. As observed in Fig 4(A), lignin composition decreased after ACO compared to ALO. The high reduction of lignin in ACO may be due to the solubility of lignin in HCOOH and the presence of HCOOOH [61]. The decomposition of HCOOOH generated HCOOH and hydroxonium ions. These ions are particularly reactive toward the aromatic chains present in lignin, leading to lignin degradation. This is followed by an increase in the number of phenolic hydroxyl groups, thus rendering the lignin more soluble in HCOOH and water. The effect of acidic extraction was confirmed by determining the lignin-to-cellulose ratio of the solid fraction. The band area at 1504–1507 cm^-1^ is assigned to the total content of lignin (aromatic skeleton vibration) and the band at 1371–1372 cm^-1^ represents the C-H in cellulose(45). Table 3 shows the lignin-to-cellulose ratio (R) of the feedstock and solid OPEFB after each alkaline and acidic extraction (Raw, ALO-1, ACO-1, ALO-2, and ACO-2). The R value of 1.82 was obtained for the raw OPEFB due to the high lignin amount in the feedstock. However, the ratio decreased from 1.43 to 1.26 after the ALO-1 and ACO-1, respectively. The R value further decreased to 0.62 after the ALO-2 and 0.53 after the ACO-2. This finding proves that the decrease of lignin content is caused by the disruption and disintegration of the functional structure of OPEFB.

**Table 3:** Ratio of lignin to cellulose (R) of Raw OPEFB and solid fraction C1AL, C1AC, C2AL and C2AC.

Samples	R (A_1504-1507_/A_1371-1374_)
Raw OPEFB	1.82
C1AL	1.43
C1AC	1.26
C2AL	0.62
C2AC	0.53

Cellulose is insoluble in water as the hydroxyl groups in sugar chains are bonded to each other, creating a hydrophobic condition. For this reason, the crystalline domain of microfibril cellulose is a hindrance to hydrolysis accessibility for nanocellulose synthesis due to the presence of extensive intermolecular hydrogen bond and van der Waals force. In this chemical system, only the cellulosic chains exposed on the surface of microfibrils are easily accessible to solvents, reactants, and chemicals. Thus, the reactivity of cellulose toward hydrolysis is very low. To improve the performance of cellulose depolymerization for nanocellulose synthesis, the supramolecular structure of cellulose should be disrupted; hence, some crystalline domains should be converted into amorphous phases [2].

Other acid reactions may apply different mechanisms towards lignocellulose valorization. Sulfuric acid is a strong acid that is commonly used in biomass pretreatment which completely dissociates in water to form hydrogen ions (H^+^) and anions to break down the lignocellulosic structure of the biomass. The mechanism cleaves the ester linkages between lignin and hemicellulose, which makes the cellulose more accessible. According to several research, concentrations of sulfuric acid beyond 65 w/w% result in cellulose swelling and structural breakdown by breaking hydrogen bonds and produce various complexes, including partial esterification of hydroxyl groups followed by their substitution by sulfate groups. Besides that, in cases where increased crystallinity is needed, the use of weaker acids would be beneficial in obtaining cellulose crystals with specific properties [62]. Another study published the effect of acid pretreatment on the primary products of biomass pyrolysis and found that formic acid and sulfuric acid pretreatment resulted in different primary products of biomass pyrolysis. Formic acid pretreatment resulted in higher yields of levoglucosan, while sulfuric acid pretreatment resulted in higher yields of furfural and 5-hydroxymethylfurfural [63].

### Functional group analysis by FTIR

**Fig 6:**
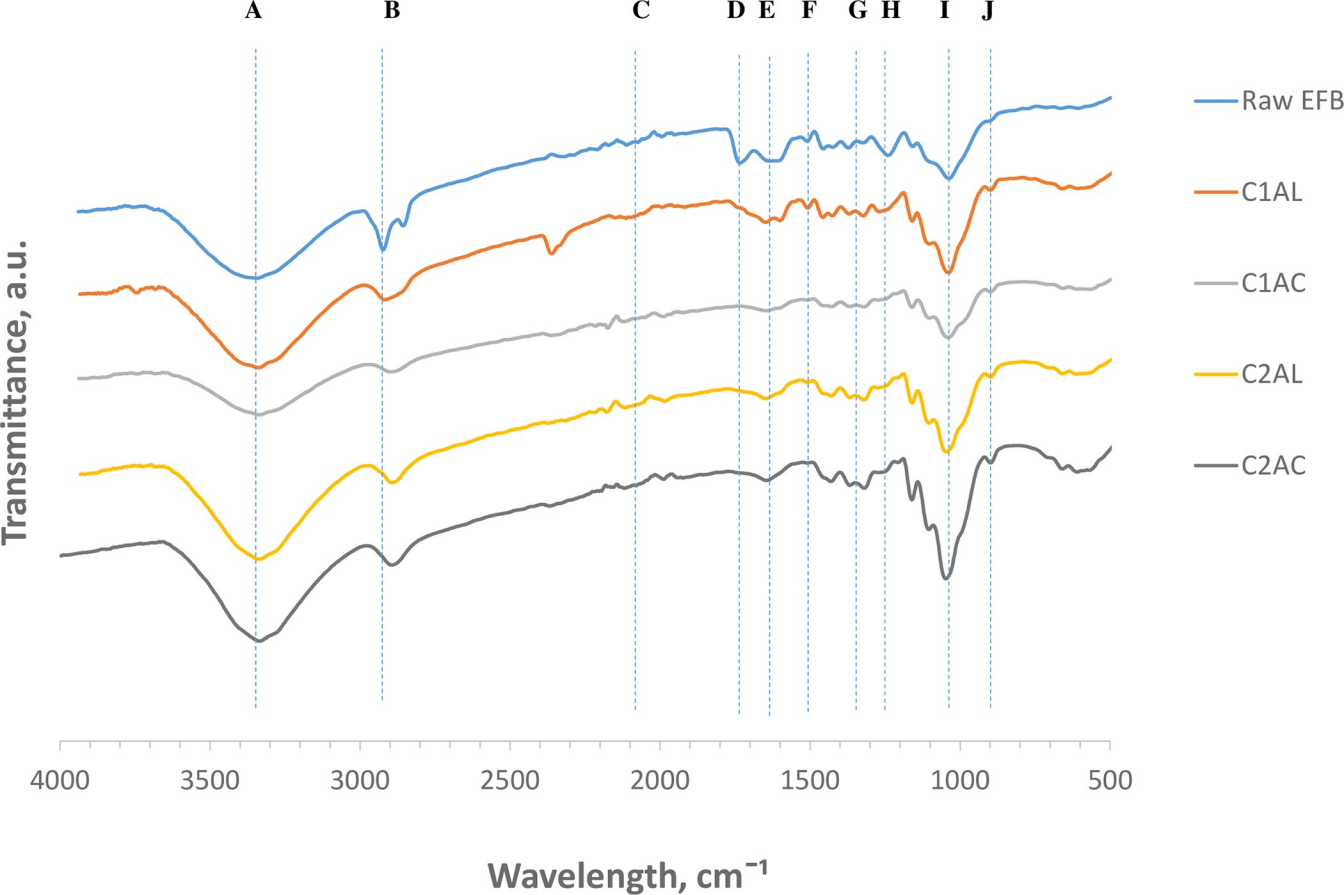
FTIR spectrum of the Raw OPEFB and extracted solid C1AL, C1AC, C2AL and C2AC.

The structural changes of raw OPEFB, C1AL, C1AC, C2AL, and C2AC were analyzed based on the FTIR spectra analysis (Fig 6). Ten peaks were identified from the spectra at wavelengths ranging from 3500 to 899 cm^-1^ and labeled as Peak A to J. The peak at a certain range of wavelengths is attributed to specific molecular groups. Comparison has been made on the intensity of the adsorption at each peak detection for different samples. Peak intensity strength was compared between samples based on the peak height ratio. In FTIR, an increase in the peak intensity usually means an increase in the amount (per unit volume) of the functional group associated with the molecular bond, whereas a shift in peak position usually means the hybridization state or electron distribution in the molecular bond has changed [64].

Table 4 shows the listed peaks, band assignments based on the literature, and the characteristics of the corresponding intensity strength for each sample, which are designated as ‘Increase’ (↑), ‘Decrease’ (↓), ‘Maintain’ (↑↓), or ‘Diminished’ (o). The spectra of raw OPEFB were set as control. Peak D, F, and H showed significant reduction after the first alkaline extraction. The band at Peak D at a wavelength around 1740 cm^-1^ is due to the unconjugated carbonyl of xylan (C = O). This peak also refers to the linkages that exist between lignin and hemicellulose. It was found that the peak diminished after the ALO-1. Peak F at wavenumber 1500 cm^-1^ is attributed to the polymer structure of aromatic lignin group C = C. Peak F and H decreased after the ALO-1 and then diminished upon the ACO-2. Peak H at wavenumber 1220 cm^-1^ is attributed to the deformation of syringyl and guaiacyl lignin. This suggests that these compounds are mostly solubilized after the first SOE cycle. The infrared intensity at these three wavenumbers decreased permanently after the first SOE cycle, which is interrelated to changes in hemicellulose and lignin structures and partial removal from the solid fraction OPEFB. The wavenumber around 3200–3500 cm^-1^ (Peak A) represents a strong and broad absorption band. In the literature, the wavenumber is assigned to O-H stretching, specifically the O-H stretching region from cellulose I [65,66].

**Table 4:** Absorption peaks detected and the corresponding functional groups of FTIR analysis for C1AL, C1AC, C2AL and C2AC according to literature.

	Peak (cm^-1^)	Functional Group Assignment	Assign polymer	Intensity strength: Increase = ↑, Decrease = ↓, Maintain = ↑↓, Diminished = O					References
				Raw OPEFB	C1AL	C1AC	C2AL	C2AC	
A	3500–3200	O-H (H-bond)	C	Control	↑	↓	↑	↑	[67,68]
B	2918	C-H	C, H. L	Control	↑	↓	↑	↓	[67–69]
C	2100	Transition of methyl carbonyl	C, H, L	Control	↑	O	↑	O	[70]
D	1750–1720	C = O (saturated aldehyde)	H, L	Control	O	O	O	O	[68]
E	1649–1652	Unordered random coils and turns of amide I. C = O, C = N, N-H	L, C	Control	↑	↓	↑	↑	[67,69,70]
F	1507–1504	C = C (in ring) aromatic skeletal	L	Control	↓	O	O	O	[68]
G	1374–1371	C-H vibration of -CH3 and -CH2	C	Control	↑	↑↓	↑↓	↑↓	[10,67]
H	1300–1200	Aromatic rings of guaiacyl and syringyl units	L	Control	↓	O	O	O	[67]
I	1200–1100	C-O-C, C-O vibration in (C6)	C, H	Control	↑	↓	↑	↑	[67,71]
J	913	C-H, amorphous area	C	Control	↑	↑↓	↑↓	↑↓	[71]

Fig 6 shows a higher intensity peak of the extracted solid after the ALO-1 (C1AL sample) at this wavenumber compared to raw OPEFB. However, the intensity peak decreased after the ACO-1 (C1AC sample). Interestingly, the peak intensity increased again after the ALO-2 (C2AL sample) and decreased after the ACO-2 (C2AC sample). This condition was also reported by Isroi et al. (2012), where the intensity of O-H stretching at the corresponding wavenumber of acid-pretreated OPEFB was lower than the untreated OPEFB [66]. Peak B at wavenumbers 2850–2900 cm^-1^ is assigned to cellulose deformation. The intensity of these bands dropped after the ALO-1, indicating the shift of the OPEFB peak. A different intensity was observed at wavenumbers 1100–1200 cm^-1^ (Peak I) assigned to the C-O-C and C-O vibration in cellulose and hemicellulose. The intensity at this wavenumber for C1AL sample increased after the ALO-1, but the intensity decreased after the ACO-1 (C1AC sample). Interestingly, the peak increased again after the ALO-2 and dropped after the ACO-2. The sharp bands at 913 cm^-1^ are attributed to β-glycosidic linkages between the sugar units in cellulose [72]. All three spectra profiles are clearly exhibited, except for the raw sample. As reported by Xu et al. (2013), the subtle band in the raw sample is probably due to the coverage of cellulose by hemicellulose and lignin. Amorphous cellulose is (1→4)-β-D-glucan [73].

### Nuclear magnetic resonance (NMR) analysis

**Fig 7:**
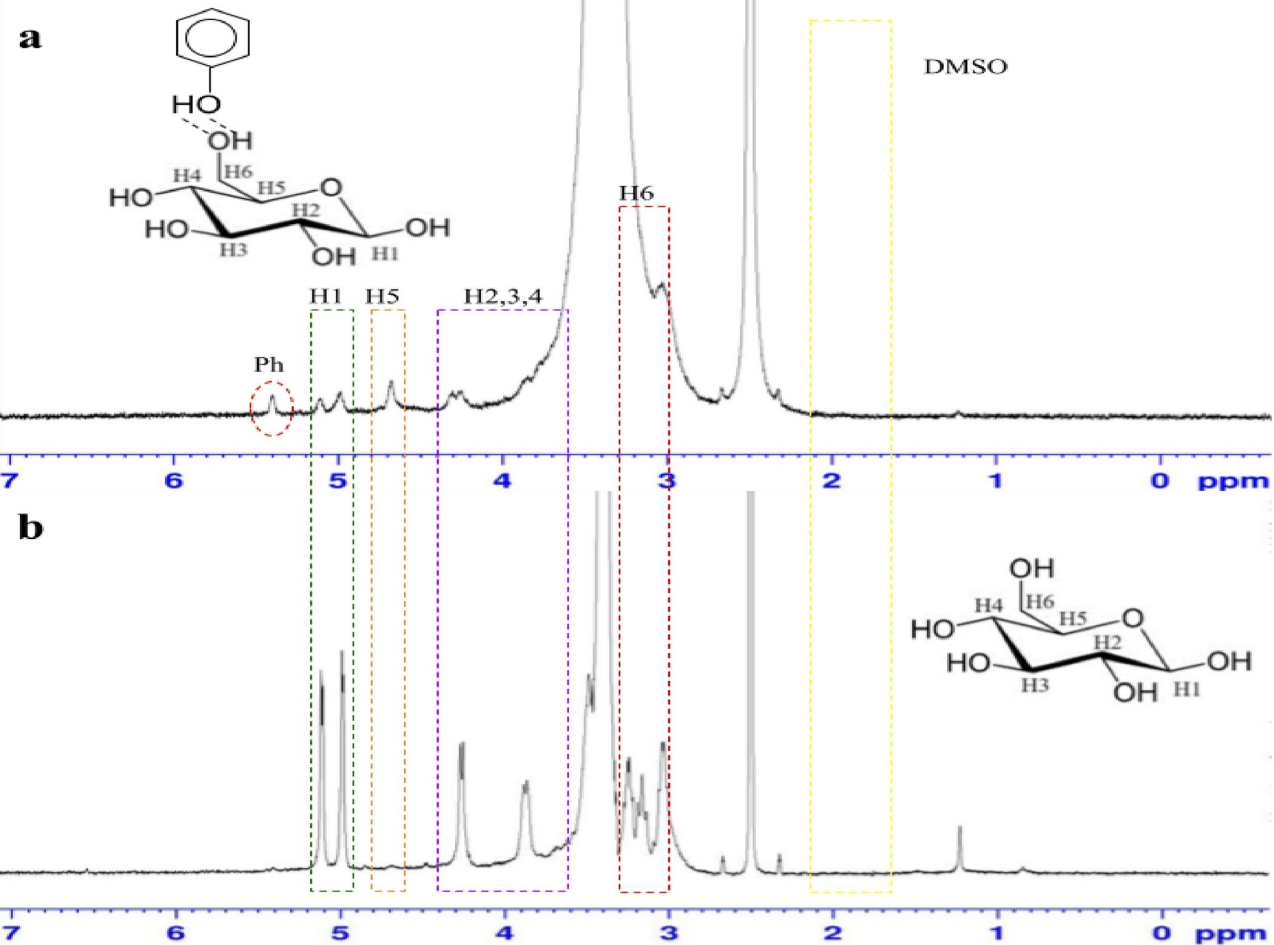
^1^H NMR spectra of extracted solid OPEFB in DMSO suspension (a) extracted solid after SOE1 and (b) SOE2.

^1^H NMR spectroscopy was used to further analyze the chemical structure of the obtained ES after the SOE-1 and SOE-2. The samples were dissolved in DMSO and analyzed at 11.74 T (400 MHz for ^1^H resonance) to determine the structure of the compound based on the type of proton or hydrogen. Fig 7 shown absorption peak that appears in the H-NMR spectrum in units of ppm or chemical shift (δ). The peaks between 5.0 and 3.0 ppm represented the protons of the cellulose backbone [74–76]. In the present study as Table 5, the peaks attributed to β (1–4) D-glucose structure (monomer of cellulose) after SOE1 are δ 4.75 (β H1), 3.60–4.42 (H2,3,4), 4.67 (H5), and 3.02 (H6) while 4.99 (β H1), 3.50–4.42 (H2,3,4), 4.71 (H5), and 3.00–3.33 (H6) for the ES after SOE2. These assigned peaks are in close approximation to reported shift value in dissolved cellulose from cotton, bleached kraft pulp [75] and rice straw cellulose [76]. The sample from SOE1 shown signal at δ 5.40 but not present in sample prior the SOE 1. The peak is assigned to proton from hydroxyl of aryl group, could be assigned to phenol structure [77] indicated the presence of lignin in the sample.

**Table 5:** ^1^H NMR analysis of OPEFB sample after SOE1 and SOE2.

^1^H NMR analysis of OPEFB sample after SOE1					
Chemical shift, δ (ppm)	Integration	^1^H number	Implication	Compound	
5.40	1.00	1	-OH of aryl	Phenol-OH
4.75	1.62	1	O-CH-OH	β H1	
4.67	1.02	1	O-CH-OH	H5	
3.60–4.42	1.31	1	R-CH-OH	H2, H3, H4	
3.02	1.99	2	R-CH_2_	H6	
2.30–2.70	-	-	DMSO	DMSO	
^1^H NMR analysis of OPEFB extracted solid after SOE2					
Chemical shift, δ (ppm)	Integration	^1^H number		Compound	
4.99	1.06	1	O-CH-OH	β H1
4.71	1.02	1	O-CH-OH	H5
3.60–4.42	1.12	1	R-CH-OH	H2, H3, H4
3.00–3.33	2.15	2	R-CH_2_	H6
2.30–2.70	-	-	DMSO	DMSO

### Physical analysis of extracted solid

#### Fiber properties and surface morphology

**Fig 8:**
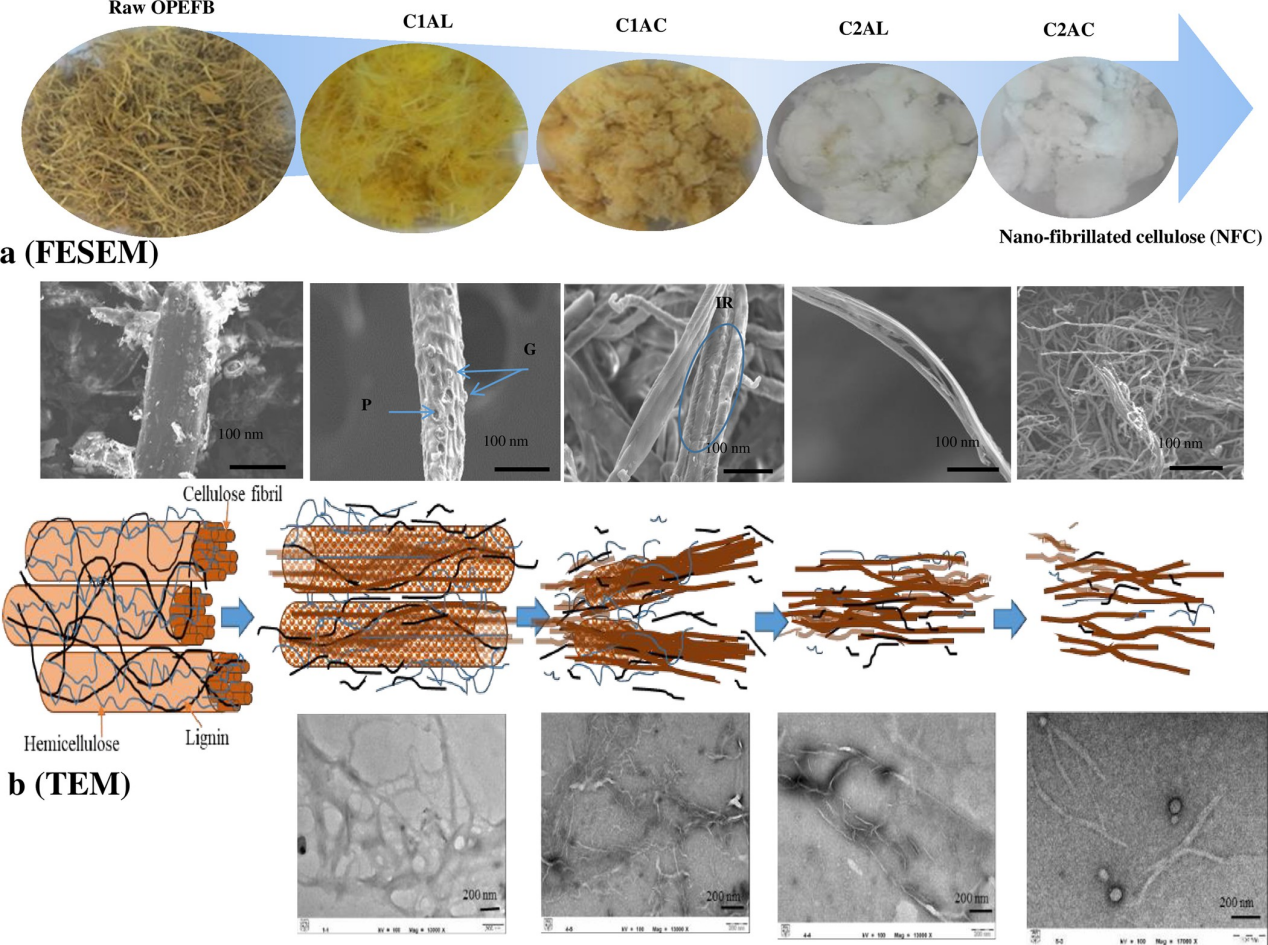
Fiber properties and surface morphology of the extracted solid C1AL, C1AC, C2AL and C2AC, analyzed using (a) FESEM and (b) TEM.

**Fig 9:**
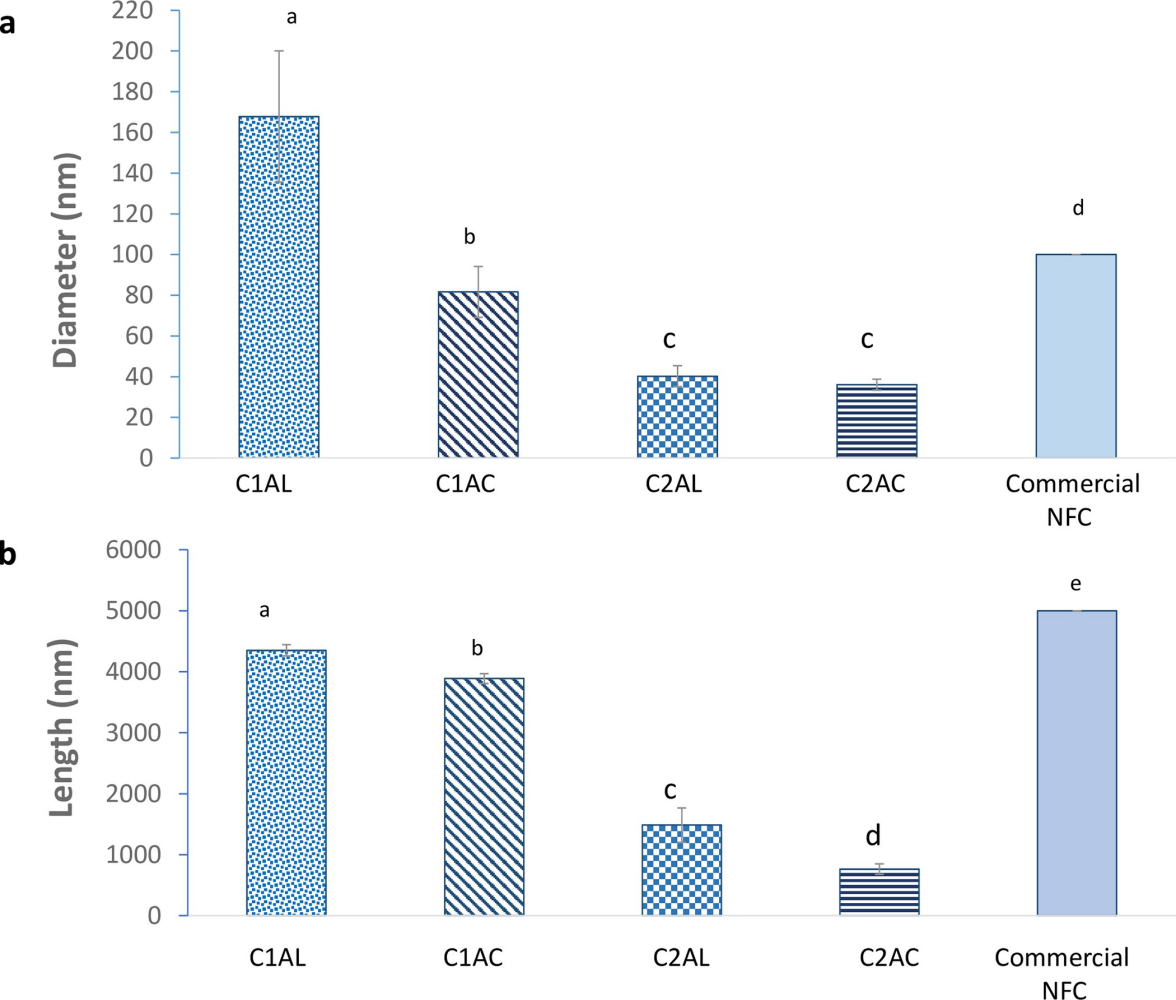
(a) Diameter and (b) length measurement of the extracted solid C1AL, C1AC, C2AL, C2AC and commercial NFC as reference. The diameter and length data shown are the mean data (n = 3) with error bars representing standard deviation. ^abc^ represents the significance difference between samples from one-way ANOVA (Turkey method, 95% confidence). Different letters indicate significantly different.

Visual characteristics of the solid fiber isolated from OPEFB (Fig 8) were presented based on the analysis of field emission scanning electron microscopy (FESEM) (Fig 8A) and TEM (Fig 8B). The diameter and length measurement for each sample is reported and commercial NFC was used as a reference (Fig 9). The FESEM analysis focuses on the surface of the sample while the TEM analysis shows the transmission of internal composition. Fig 8A indicates that raw OPEFB is a rigid and compact composite, in which the external surface consists of heavy deposition of wax, hemicellulose, lignin, and other inorganic components. Upon the ALO-1, the fibers changed from brownish to yellowish.

As the observation of the surface morphology of the extracted solid C1AL, the solid structure contains multiple pores (labeled as P) and remarkable globular structures are present (labeled as G). It is believed that the globular structure is associated with the agglomeration of lignin on the surface due to solubilization, which then coalesces to form droplets that come into contact with the cell wall matrix. Some fraction of the lignin is forced to the outer surface due to the hydrostatic pressure within the cell wall layers [74]. These conditions indicate that lignin experienced severe stripping as ALO disrupts the lignin structure and breaks the linkage between lignin and other carbohydrate fractions in the lignocellulosic biomass. The specific surface area increases after the ALO-1, making the biomass more exposed and accessible.

As per the TEM image, there is some evidence of the swelling effect, where some parts of the solid OPEFB distanced and produced fibrillar parts isolated from each other; subsequently, a network-like structure was formed with branched morphology. The size also varies, resulting in a high standard deviation of fiber diameter. There are multiple smaller elementary fibrils at the fiber ends, with bulk and untangled fiber ends on the other part. The diameter of the fiber varies from 140 to 200 nm and the length is approximately 4,200–4,400 nm; hence, the fiber can be classified as MFC.

Upon the ACO-1, the surface image morphology of the fiber (C1AC) shows a smoother surface fiber, but some irregular surface (labeled as IR) is still present. The TEM image of this sample shows that some parts of the fiber have a more transparent effect compared to others, with higher dissociated fibrils could be observed. The more transparent fiber image indicates the presence of thinner lignin and hemicellulose on the solid [75] supporting that the solubilization of non-cellulosic components occurred during ACO. The image also portrays more untangled fibrils with a smaller diameter, which is around 70–95 nm with a small reduction in length (3,800–4,000 nm) compared to C1AL. Additionally, the dimension of the extracted solid fiber after the first SOE cycle is less than the commercial NFC. Therefore, dimensionally, OPEFB fiber can be classified as NFC after completing the first SOE cycle. However, its yellowish color and lignin content may become other aspects to be considered. The second SOE cycle, particularly after the ALO-2, resulted in whitish fiber, and the SEM image shows a smooth fiber without any irregular surface. This may indicate the removal of most of the lignin and other non-cellulosic components from the OPEFB fiber after the second ALO. Based on the TEM image captured for the non-network fiber structure, it could be identified that most of the fibrils were untangled and detached from each other, resulting in a smaller fiber size with 35–45 nm in diameter. On the other hand, the fiber length varies from 1,400 to 1,500 nm, leading to a higher standard deviation. It may be because the ALO-2 produces fibrillar parts and solubilizes amorphous cellulose, resulting in the shortening of the fiber.

After the ACO-2, the fiber appeared snow-white color and was significantly shorter (670–850 nm) but the change of diameter was less significant (33–38 nm) compared to the fiber after the ALO-2. This outcome shows that the ACO-2 affects more on the shortening of the fiber as most of the non-cellulosic components have already been removed after the ALO-1. However, the fiber is still classified as NFC rather than NCC because its length exceeds 500 nm and it has a fibril shape rather than a needle-like structure.

#### Crystallinity index

**Fig 10:**
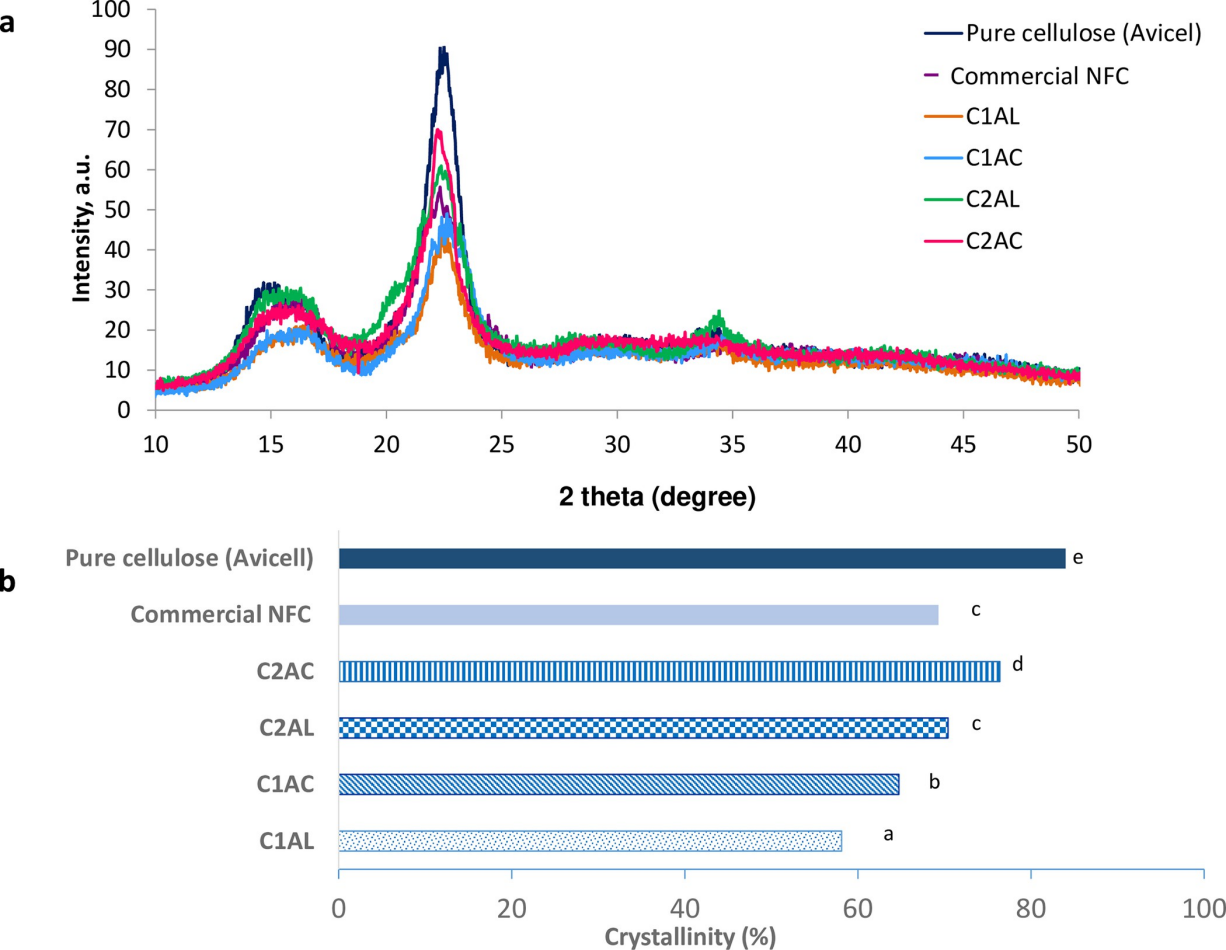
(a) XRD diffractogram (b) Crystallinity index (%) as the ratio of crystalline and amorphous peaks of the extracted solids C1AL, C1AC, C2AL, C2AC compared with pure cellulose (Avicel) and commercial CNF. ^abc^ represents the significant difference between samples. Different letters indicate significantly different.

The crystallinity index (CrI) of all extracted solid fibers were analyzed by the X-ray diffraction (XRD) patterns. The CrI specifies the ratio of crystalline to amorphous regions of cellulose. The properties of cellulose crystallinity have been used to identify the physical characteristics of fibers [76]. An increase in crystallinity is expected to increase the stiffness and rigidity, therefore providing better strength, and conferring a higher resistance to cracks. Fig 9A compares the XRD diffractogram of the extracted solids C1AL, C1AC, C2AL, C2AC with pure cellulose (Avicel®101) and commercial NFC. All the samples exhibited high peak intensity at 2θ = 22°, which is related to the crystalline structure of cellulose. The presence of a broad peak at around 2θ = 15° is related to the amorphous arrangement [76]. The CrI was calculated as the ratio of crystalline and amorphous peaks and the results are summarized in (Fig 10). Upon the ALO-1, the C1AL sample recorded the CrI of 58.12% as it still has many amorphous regions. After the ACO-1, the CrI of the C1AC sample increased to 64.73% due to further removal of hemicellulose and lignin from the fiber. An increased CrI was observed for the extracted fiber upon the second SOE cycle of the ALO-2 and ACO-2 with 70.41% and 76.42%, respectively. The increase of CrI after the second SOE cycle indicates the dissolution of the amorphous region of cellulose, allowing the hydrolytic cleavage of glycosidic bonds and eventually shortening the cellulose chain. Meanwhile, the CrI of the commercial CNF was 69.3%, whereas the CrI of pure cellulose (Avicel® 101) was 84%. Therefore, the CrI of the extracted fiber upon the ALO-2 and ACO-2 is higher than that of the commercial CNF but lower than the CrI of pure cellulose.

#### Water absorption

**Fig 11:**
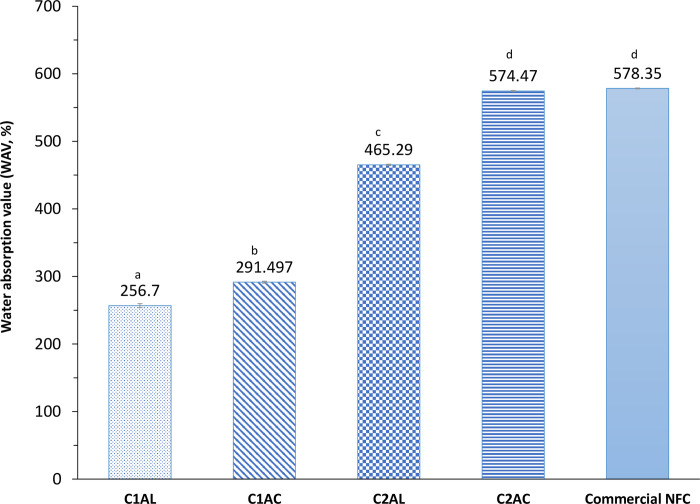
Water absorption number (WAN) of extracted solids C1AL, C1AC, C2AL, C2AC compared with commercial CNF. The WRN data shown are the mean data (n = 3) with error bars representing standard deviation. ^abc^ represents the significant difference between samples. Different letters indicate significantly different.

The value of water absorption or water holding capacity indicates the ability of the fiber to absorb water and swell. When the extracted solid fiber is dispersed in water, the fibrils trap water between them and do not release the water easily, hence preventing the free flow of water. The properties are closely related to the surface morphology and bonding ability of the extracted fiber [78]. The results (Fig 11) show that the water absorption number (WAN) of extracted solids increased as the SOE progressed from the ALO-1 to the ACO-1, ALO-2, and ACO-2. Throughout the processes, the fibrillation of raw OPEFB occurred, where cellulose fibers disintegrate into thinner fibrils, as illustrated in Fig 8. At the same time, the surface area increased as the bundled raw OPEFB cellulose is untangled to nanofibrils. Another important factor for increasing water absorption is the special interaction between the cellulose fibril surface and water. The fiber mainly consists of cellulose, which has many hydroxyl groups and forms strong hydrogen bonds with water molecules, thus preventing them from bonding on the fiber surface [78]. As the surface area increased, more hydroxyl groups are available, thus, the water absorption increased.

#### Zeta potential

**Fig 12:**
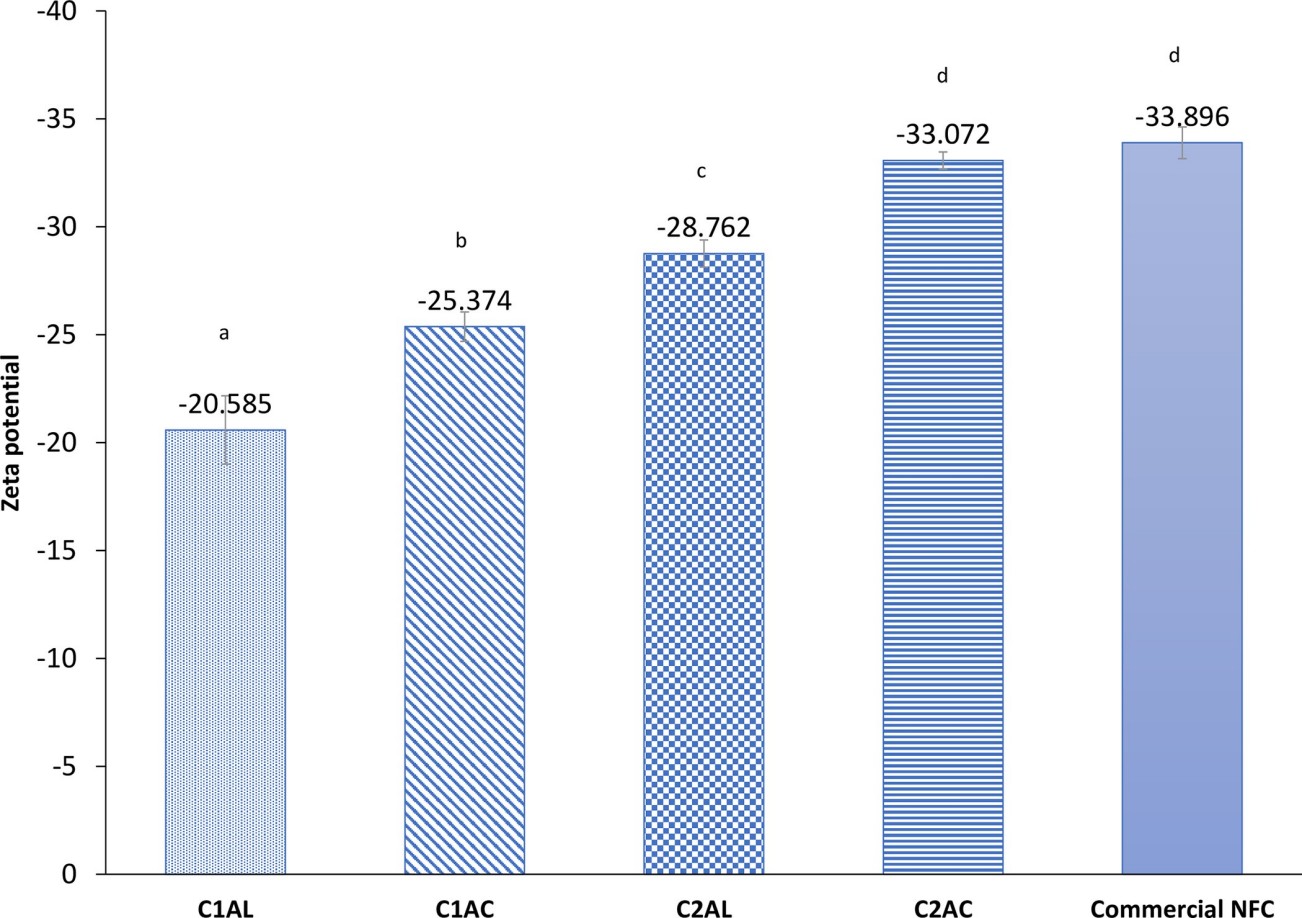
Zeta potential of extracted solids C1AL, C1AC, C2AL, C2AC compared with commercial CNF. The zeta potential data shown are the mean data (n = 3) with error bars representing standard deviation. ^abc^ represents the significant difference between samples. Different letters indicate significantly different.

The zeta potential is the evaluation of the stability and dispersion of fibers/extracted solids in an aqueous solution. The fiber solution presented a negative zeta potential (Fig 12) due to the deprotonation of carboxylic acid throughout the oxidation process. This phenomenon provides the repulsion force between individual fibers, leading to uniform and stable dispersion in aqueous media [79]. The nanocellulose after the second SOE (C2AC) were found to have a significantly higher negative surface charge (-32.57 mV) compared to extracted cellulose after first SOE (-28.762 mV). The zeta potential results of the nanocellulose from this study was consistent with commercial CNF (-33.896) and another study where the CNF was isolated by alkali and TEMPO oxidation reported a very stable suspension of nanocellulose in water [31,79].

#### Thermogravimetric analysis (TGA)

**Fig 13:**
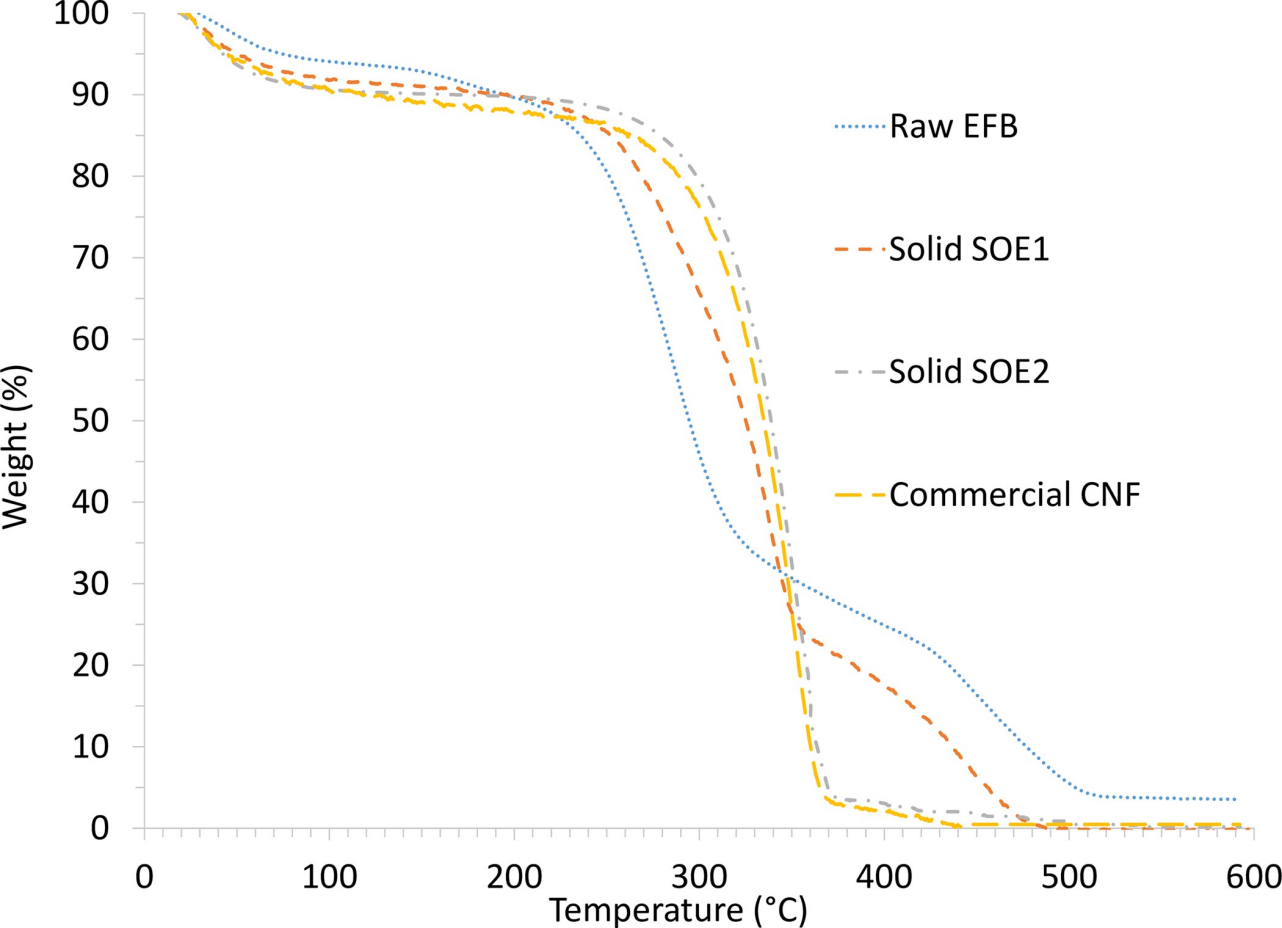
TGA thermograms curves of the raw OPEFB, extracted solid fiber of SOE1 and SOE2 and commercial CNF.

Thermal stability of the obtained extracted solid upon SOE1 and SOE2 was identified by thermogravimetric analysis (TGA), correspond to the weight loss of the samples upon continuous heating to 600˚C. Fig 13 presents the TGA thermograms curves of the raw OPEFB, extracted solid fiber of SOE1 and SOE2 and commercial CNF. Lignin decomposes over a broad range of temperatures (150–900˚C), while generally, hemicellulose decomposes at temperature range 220–315°C and cellulose at 300–400°C [80,81]. An initial weight loss of the fibers occurs below 100˚C is ascribed to the water vaporization which was dependent on the initial moisture content of the analyzed sample. At higher temperatures, as shown in Fig 13, a sharper weight reduction is observed. It was identified that, at temperature of 150˚C, 204˚C, 232˚C, 242˚C for raw OPEFB, extracted fiber SOE1, commercial NFC and extracted fiber SOE2 respectively was supposed as the starting decomposition temperature of the samples. The order of the starting decomposition temperature increased in the trend of raw OPEFB < extracted fiber SOE1 < commercial CNF < extracted fiber SOE2. As in this study, raw OPEFB showed weight loss start at temperature 150˚C indicated the presence of lignin in the sample. Compared to hemicellulose and cellulose, lignin decomposes earlier because its structure constitutes of phenol and aromatic rings with various branches. The activity of the chemical bonds in lignin covered a wide range, thus leading to the degradation of lignin occurring in a wide temperature range [81,82]. This was verified as in Fig 13, significant weight loss was identified above temperature 400˚C for raw OPEFB and fiber SOE1 samples as lignin is contained in this samples. Removal of lignin from the OPEFB as extracted fiber SOE1 and SOE2 improves the sample’s thermal stability. Their weight loss mainly happens at temperature above 220˚C as designated the decomposed of hemicellulose [82]. Cellulose decomposition was focused at a higher temperature range (313–400), and when the temperature was higher than 400, most of the cellulose was pyrolyzed [82,83]. Extracted fiber SOE2 was identified to have higher decomposition temperature compared to commercial NFC may because the SOE2 fiber contained less lignin and hemicellulose besides higher crystallinity index as reported above (Fig 10).

## Conclusion

Sequential oxidative extraction (SOE) consists of alkaline and acidic processes assisted by H_2_O_2_ as an oxidation agent. In this study, alkaline and acidic conditions were prepared using a low concentration of NH_4_OH and HCOOH. The effects of SOE on the OPEFB extracted solid and liquid chemical compositions, structural changes, size and surface morphology, degree of crystallinity, water retention, zeta potential and TGA were studied. As the SOE progressed in a sequence of ALO-1, ACO-1, ALO-2, and ACO-2, the lignin and hemicellulose content from the extracted solid decreased, leading to an increase in the cellulose content in the fiber. Material balance illustrated two SOE cycles successfully removed 99.4% lignin and 89.8% hemicellulose from the OPEFB and produced fiber with 78.58% cellulose. Based on the study on the size and surface morphology of the extracted fiber through FESEM and TEM, it is determined that each stage of SOE (ALO-1, ACO-1, ALO-2, and ACO-2) produced fibers with various dimensions, and interestingly, different fiber dimensions provide different potential applications. The increase of CrI upon the second SOE cycle indicates the dissolution of the amorphous region of cellulose, allowing the hydrolytic cleavage of glycosidic bonds and eventually shortening the cellulose chain. Moreover, the fiber solution recorded a negative zeta potential, leading to uniform and stable dispersion in aqueous media.
